# Serum levels of Homocysteine, Vitamin B12 and Folate in Patients with Multiple Sclerosis: an Updated Meta-Analysis

**DOI:** 10.7150/ijms.42058

**Published:** 2020-03-05

**Authors:** Xuanting Li, Junliang Yuan, Jinming Han, Wenli Hu

**Affiliations:** 1 Department of Neurology, Beijing Chaoyang Hospital, Capital Medical University, 100020, Beijing, China.; 2 Department of Clinical Neuroscience, Karolinska Institute, Stockholm, Sweden.

**Keywords:** Multiple sclerosis, Homocysteine, Vitamin B12, Folate, Meta-analysis

## Abstract

**Background**: Multiple sclerosis (MS) is a demyelinating and disabling inflammatory disease of the central nervous system. MS is triggered by complex environmental factors which mostly affect genetically the susceptible young people. Emerging data has suggested that changes of homocysteine (Hcy), Vitamin B12 and folate serum levels may be associated with MS. However, previous findings are not always consistent.

**Methods**: In this study, we aimed to investigate the relationships between MS and Hcy, Vitamin B12 and folate with updated available data (until September, 2019). The diagnosis of MS was performed based on international criteria for the diagnosis of MS, including magnetic resonance imaging and cerebrospinal fluid tests. We searched the databases including PubMed, EMBASE, Cochrane Library and ScienceDirect. After data collection, separate analyses based on random-effect models were used to test for relationships between MS and Hcy, Vitamin B12 or folate blood levels. The effective sizes were estimated by the combined standardized mean difference (*SMD*) and associated 95% confidence interval (*CI*).

**Results**: Based on the inclusion criteria, a total of 21 original studies with 1738 MS patients and 1424 controls were included in this study. There were 17 studies for measuring Hcy, 16 studies for measuring Vitamin B12 and 13 studies for measuring folate in patients with MS, respectively. Specifically, patients with MS had higher serum levels of Hcy (*SMD*: 0.64; 95% *CI*:0.33, 0.95; *P* <0.0001) compared with control groups. There were no significant differences of *SMD* for Vitamin B12 (*SMD*: -0.08; 95% *CI*: -0.35, 0.20; *P*=0.58) or folate (*SMD*: 0.07; 95% *CI*: -0.14, 0.28; *P*=0.52) between MS and controls. Subgroup analysis demonstrated that there was statistically significant difference for Hcy between relapsing-remitting MS (RRMS) patients and controls with a *SMD* of 0.67 (95% *CI*: 0.21, 1.13; *P*=0.004). However, no significant difference of Hcy serum levels between secondary progressive MS patients or primary progressive MS patients and controls was noted in this study. In addition, there was no significant difference of Hcy levels in females (*SMD*: 0.22; 95% *CI*: -0.16, 0.60; *P*=0.25) or males (*SMD*: 0.56; 95% *CI*: -0.13, 1.26;* P*=0.11) between MS patients and controls.

**Conclusions**: Higher serum levels of Hcy were noted in patients with MS when compared with control groups. And the difference was especially significant between RRMS patients and controls. Hcy may play an important role in the pathogenesis of MS. Functional studies are required to assess the effects of Hcy on patients with MS at the molecular level.

## Introduction

Multiple sclerosis (MS) is a neuro-inflammatory disease of the central nervous system (CNS), often followed by progressive and irreversible neurological dysfunction 1,2. It affects more than two million young individuals worldwide and imposes an enormous economic burden on the societies 3. To date, the pathogenesis of MS has been not yet well defined and there are always uncertainties regarding the future course. The combined effects of genetic predispositions such as human leukocyte antigen variants and environmental factors such as low Vitamin D levels, cigarette smoking, obesity and sun exposure on MS have been recognized recently 4,5. Understanding the potential mechanisms of MS is essential to elucidate novel treatment strategies to repair myelin and axonal structures. Several previous studies have investigated the roles of homocysteine (Hcy), Vitamin B12, and folate in MS since myelin replacement requires normal function of “folate-Vitamin B12-methylation” pathway which is vital to provide methyl groups for myelin regeneration 6. Hcy stems from methionine metabolism and can be removed either by conversion to cysteine or by remethylation to methionine, playing a physiologic role in deoxyribonucleic acid metabolism guided by folate, Vitamin B6 and B12 7. Hcy exerts direct effects on cell damage and the activation of macrophages. Increased Hcy levels in the circulation play an underestimated role in the process of MS 8. Folate and Vitamin B12 are needed in the process of methionine-synthase mediating the conversion of Hcy to methionine 9. Both 5-methyltetrahydrofolate and methyl-Vitamin-B12 are essential factors for methionine synthesis of Hcy 10. Lacking Vitamin B12 and folate may cause an increased level of Hcy.

Several previous pioneering studies have demonstrated higher Hcy levels, lower Vitamin B12 and folate levels in patients with MS compared with healthy controls 11-13. However, there are still some controversies about the final conclusions. Specifically, an earlier meta-analysis showed increased Hcy levels but lower Vitamin B12 levels in patients with MS and no significant difference of folate noted between MS and controls 14. A recent meta-analysis indicated that MS patients have elevated Hcy levels compared to controls, and no significant difference of Vitamin B12 or folate was found between the two groups 15. The latest meta-analysis published in 2019 mainly focused on Chinese population, suggesting that increased Hcy levels and reduced Vitamin B12 levels were found in Chinese MS patients 16. However, the conclusion has not been further confirmed 6,17,18, showing that there were no significant differences of serum Hcy and Vitamin B12 levels between MS patients and controls 6. Along this line, some studies showed MS patients had higher levels of Hcy which were associated with disease progression 17, but it had not been confirmed by recent studies as well 18. Due to the above inconsistent findings of serum Hcy, folate, and Vitamin B12 levels in patients with MS, we performed a novel meta-analysis to investigate serum levels of Hcy, Vitamin B12 and folate in MS patients based on updated available data.

## Materials & Methods

### Literature search

In order to explore the relationship between Hcy, Vitamin B12, folate serum levels and MS, a systematic literature research was performed in English throughout the following databases: PubMed, EMBASE, Cochrane Library, and ScienceDirect from January 1992 to September 2019. The search strategies included the following terms: “homocysteine”, “hyperhomocysteinemia”, “Vitamin B12”, “cobalamin” “folate”, “folic acid” and “multiple sclerosis”. We further examined the reference lists in case we missed some relevant articles in the initial search.

### Selection criteria

The inclusion criteria: (1) the diagnosis of MS was performed according to the criteria for the diagnosis of MS 19-24; (2) detailed data were provided by means and standard deviations (*SD*) of Hcy, Vitamin B12 and folate blood levels in patients with MS and controls, or other parameters to deduce the aforementioned values. The exclusion criteria: (1) a single case report, review, meta-analysis, letter to the editor, editorial, treatment study or proceeding; (2) studies with incomplete data; (3) studies that included patients with other neurodegenerative disorders; (4) publications that were not reported in English.

### Data collection

A standardized form was used to assess the eligibility of included articles by two neurologists. Data were collected including the first author, year of publication, study design, language of article, country, number of participants with age and sex. The mean and *SD* of concentrations of Vitamin B12, folate and Hcy in the circulation were extracted and recorded from the data tables published in each study or were calculated from other related parameters such as median and range values 23,24. Hcy, Vitamin B12, and folate concentrations with different units were standardized by Revman 5.3. A score of up to 9 points was assigned to each study according to the Newcastle-Ottawa Scale to evaluate the study quality.

## Statistical analysis

### Effect Size and Calculations

The data were analyzed using RevMan 5.3 provided by the Cochrane Collaboration. Standardized mean difference (*SMD*) was calculated for continuous variables. A 95% confidence interval (*CI*), excluding 0 or *P*<0.05, was considered statistically significant.

### Assessment of Bias

Funnel plot (RevMan 5.3) was used to assess the publication bias, Begg's tests and Egger's tests were also performed to further identify the publication bias (Stata 12.0).

### Heterogeneity

Heterogeneity of study results was assessed using a standard *I^2^* test. A fixed-effect model was used when *I^2^*<50%, while a random-effect model was used when* I^2^*>50%.

### Sensitivity analysis

We excluded studies with different study method to re-analyze the data and test the stability of this study.

## Results

### The characteristics of the included studies

**Figure 1:**
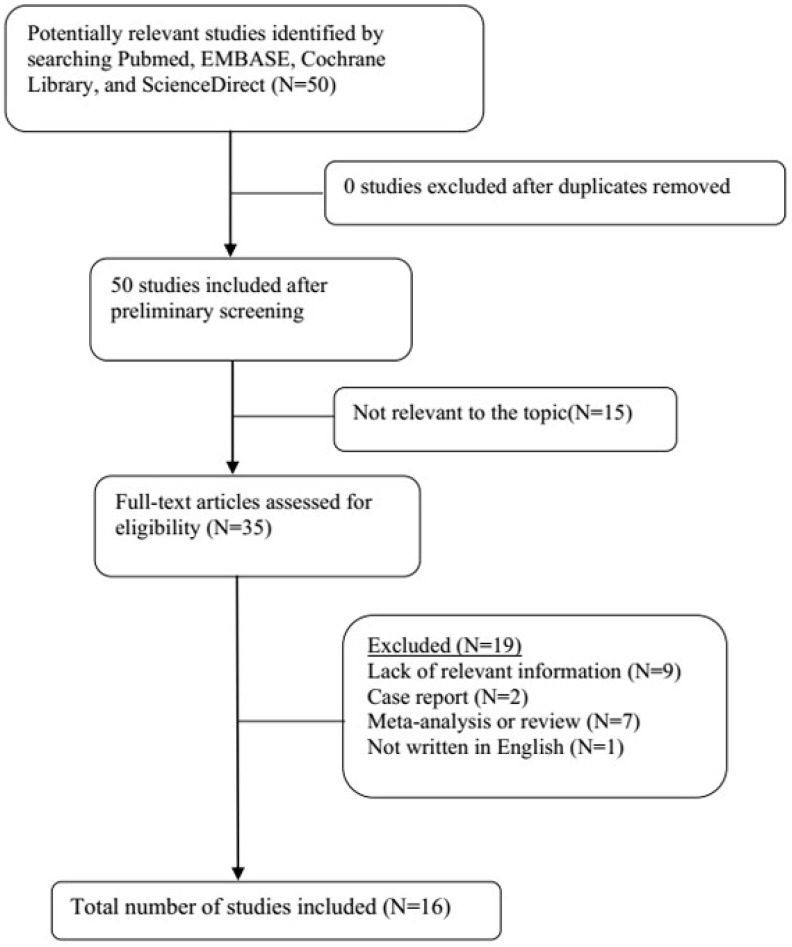
Flow diagram showing the progress of study collection for Hcy meta-analysis.

**Figure 2:**
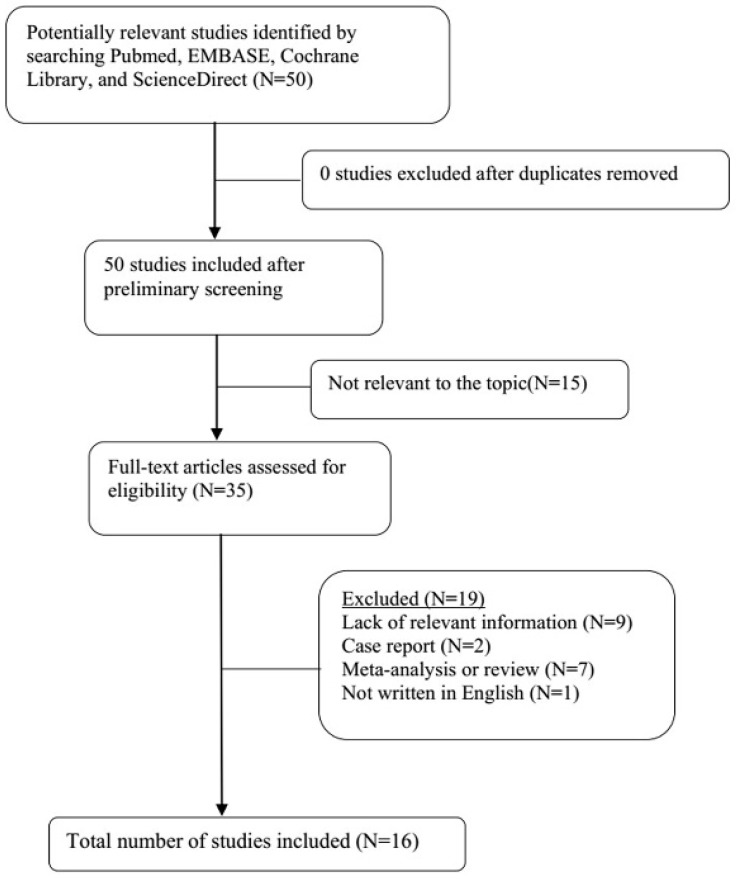
Flow diagram showing the progress of study collection for Vitamin B12 meta-analysis.

**Figure 3:**
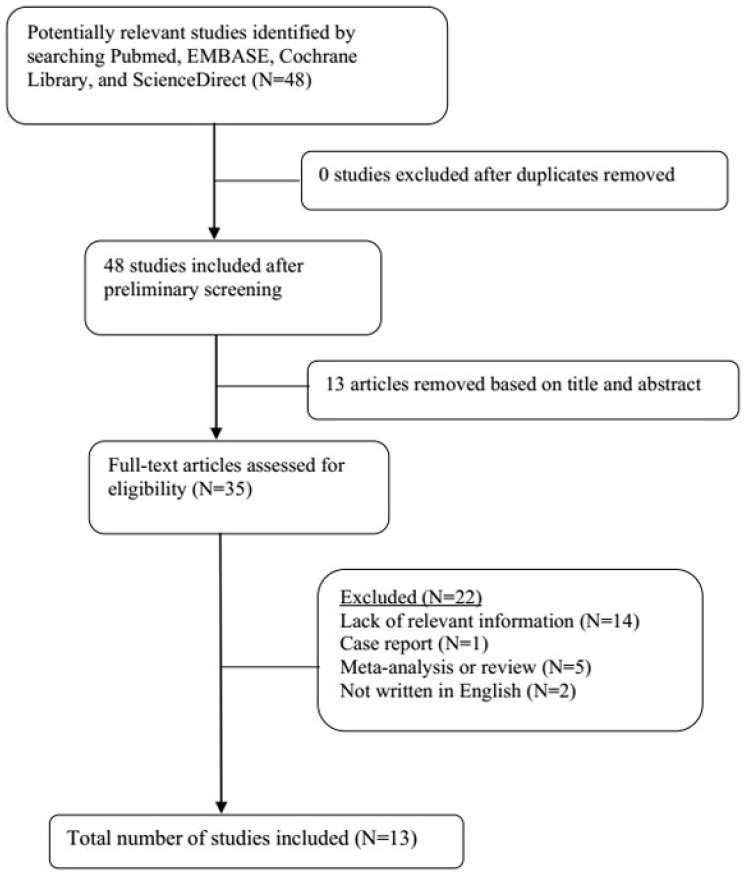
Flow diagram showing the progress of study collection for folate meta-analysis.

A total of 21 original studies were selected in our meta-analysis, including 1738 MS patients and 1424 controls. Sixty-seven studies were available for analyzing the association between Hcy and MS. Of these, 21 were removed based on the title and abstract including repetitive articles. Twenty-nine articles were excluded because they did not include related information or were not written in English. Finally, a total of 17 studies were selected according to the predefined inclusion and exclusion criteria 6,11,12, 16, 27-39. The association between Vitamin B12 and MS has been investigated by 50 different studies. Of these, 34 studies were excluded due to repetitive results or lacking related information. Finally, 16 studies met the criteria and were selected 6,12,13,16,18, 27-31, 34-36, 38,40,41. Forty-eight studies were available to explore the association between folate levels and MS, and 13 studies were included according to the predefined inclusion and exclusion criteria 6,12,13,16, 27-30, 34-36, 38. After literature quality assessment, scores of most of the involved studies were above 6 points, while 7 studies scored 5 11,41 or 6 13,28,29,40,41 points. As for the subgroup analysis, Hcy levels were divided into three groups according to different subtypes of MS, including 9 studies for relapsing-remitting MS (RRMS) 6,11,12,29,30,32,33,36,39, 5 studies for secondary progressive MS (SPMS) 6,11,29,32,36, and 3 studies for primary progressive MS (PPMS) 29,32,36. Furthermore, we analyzed Hcy levels in different gender subgroups 30,32,36,38. The flow diagrams summarizing the selection process were presented in Figs. 1-3, and the basic data of the included studies were shown in Table 1.

### Hcy and MS

**Figure 4:**
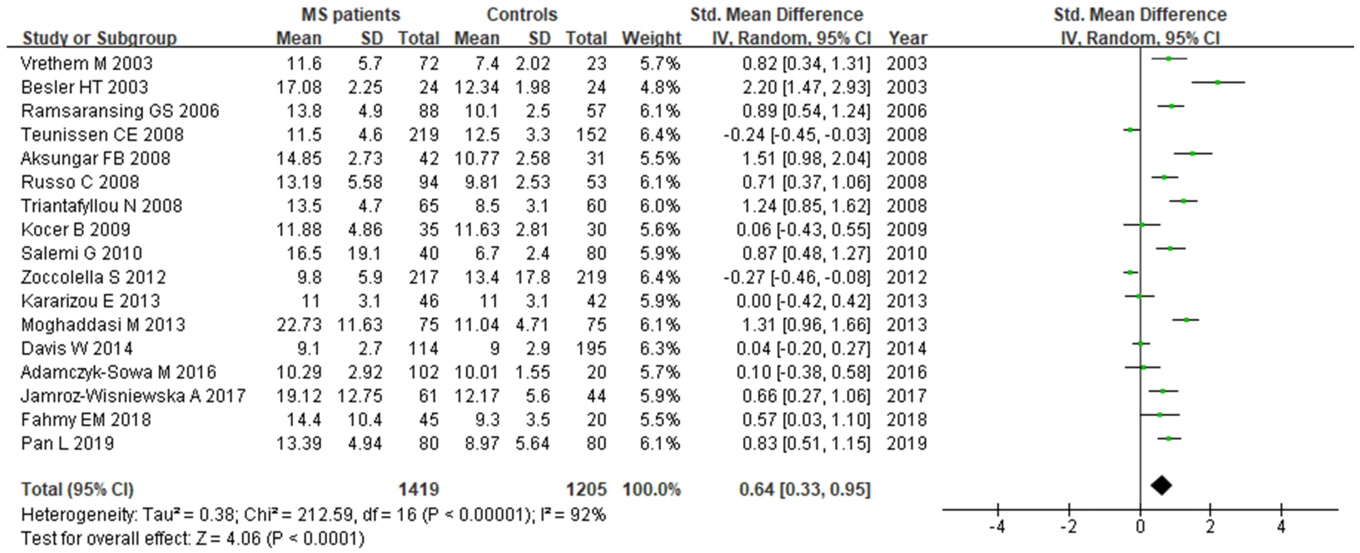
** Forest plot: *SMD* of Hcy serum levels between MS patients and controls.** Abbreviations: *SMD:* standardized mean difference; *SD*: standard deviation; Random: the random-effect model; 95% *CI*: 95% confidence interval.

**Figure 5:**
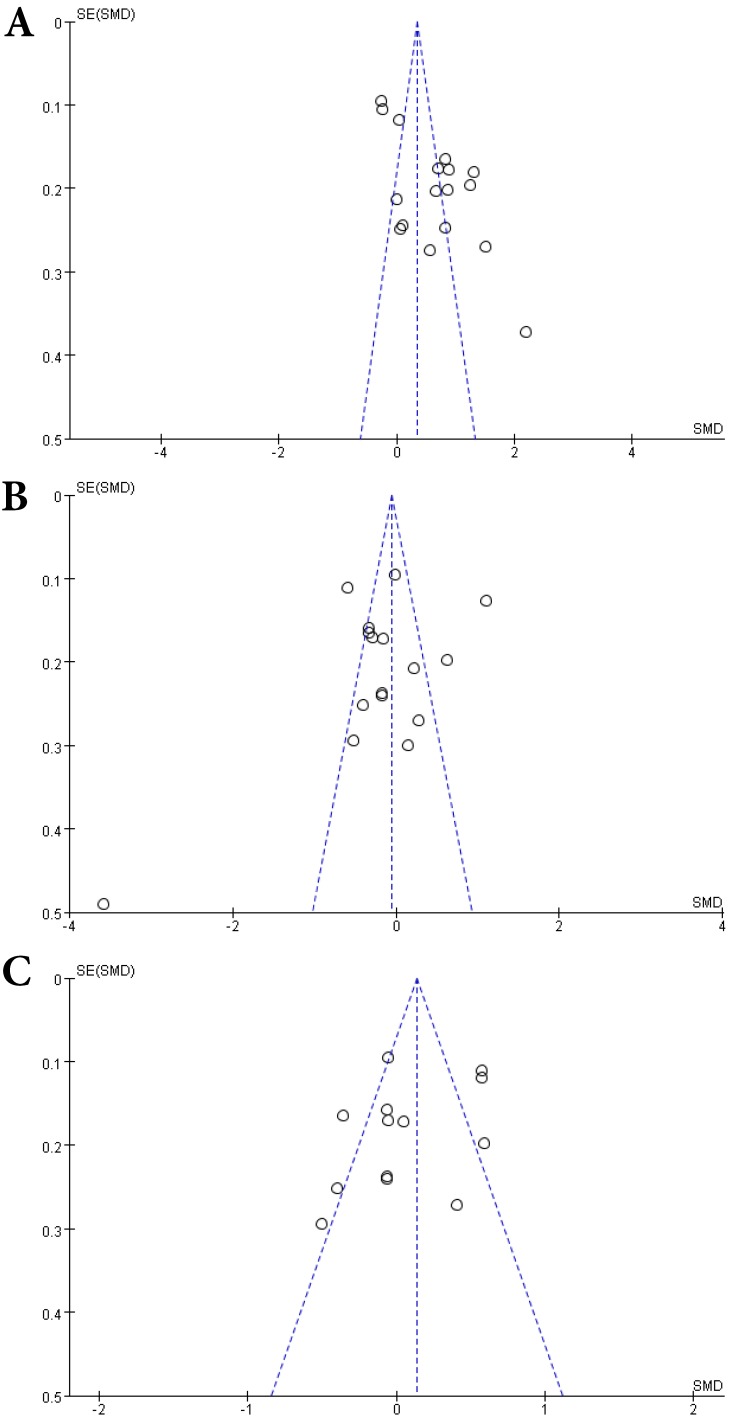
Funnel plots of comparisons of Hcy **(A)**, Vitamin B12 **(B)** and folate **(C)** between MS patients and controls.

A total of 2624 participants (1419 patients and 1205 controls) from 17 studies were eligible. A random-effect model was used with *I^2^* of 92%. Pooled results revealed a *SMD* of 0.64 (95% *CI*: 0.33, 0.95; *P*< 0.0001) (Fig. 4). This finding suggested that MS is associated with higher levels of serum Hcy. The funnel plot was symmetrical (Fig. 5A). Egger's (*P*=0.001) and Begg's (*P*=0.232) tests showed no significant publication bias for these studies, but there might be other factors effecting the symmetry of the funnel plot such as heterogeneity and sample size.

### Vitamin B12 and MS

**Figure 6:**
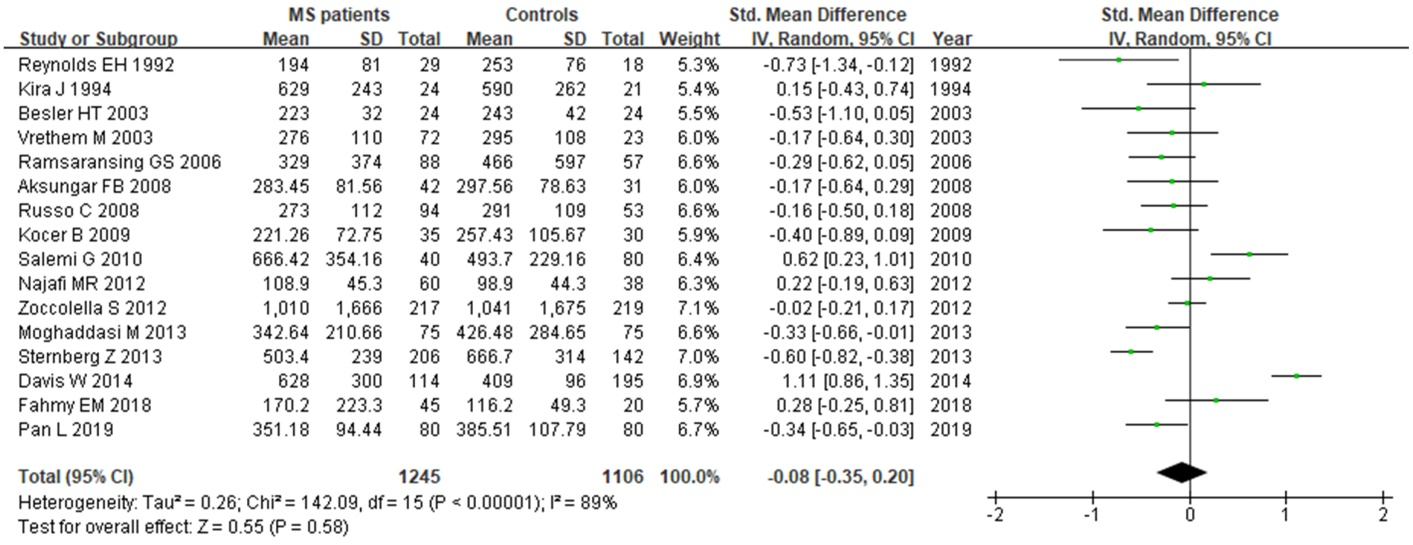
** Forest plot: *SMD* of Vitamin B12 serum levels between MS patients and controls.** *SMD:* standardized mean difference;* SD:* standard deviation; Random: the random-effect model; 95% *CI*: 95% confidence interval.

Sixteen studies describing the relationship between Vitamin B12 and MS were included with 2351 participants (1245 patients and 1106 controls). There was significant heterogeneity between selected studies for Vitamin B12 (*I^2^*=89%), so a random-effect model was utilized. The pooled findings revealed a *SMD* of -0.08 (95% *CI*: -0.35, 0.20; *P*=0.58) (Fig. 6). The symmetrical funnel plot (Fig. 5B), Egger's (*P*=0.318) and Begg's (*P*=0.964) tests all showed that there was no significant publication bias for these studies.

### Folate and MS

**Figure 7:**
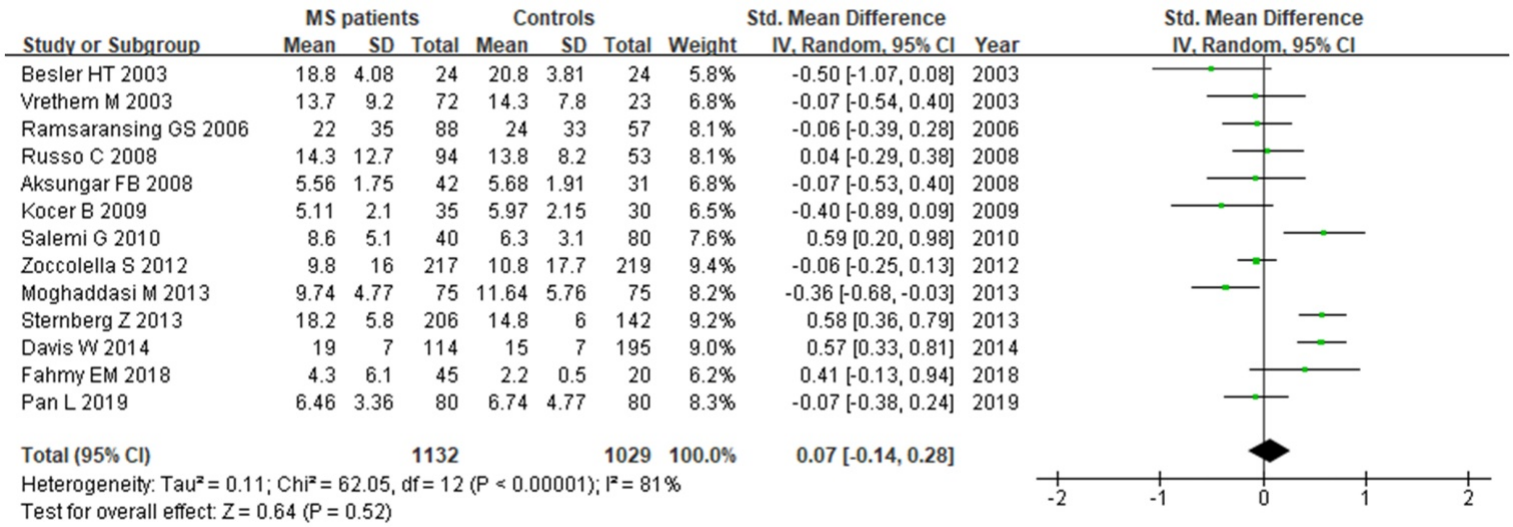
** Forest plot: *SMD* of folate serum levels between MS patients and controls.** *SMD*: standardized mean difference; *SD*: standard deviation; Random: the random-effect model; 95% *CI*: 95% confidence interval.

A total of 2161 participant (1132 patients and 1029 controls) from 13 studies were eligible. A random-effect model was applied with significant heterogeneity (*I^2^*=81%). Pooled results revealed a *SMD* of 0.07 (95% *CI*: -0.14, 0.28; *P*=0.52) (Fig. 7). The funnel plot depicting folate levels was symmetrical, indicating no publication bias (Fig. 5C). The *P* values of Begg's and Egger's tests were 0.760 and 0.262, respectively.

### Subgroup analysis

**Figure 8:**
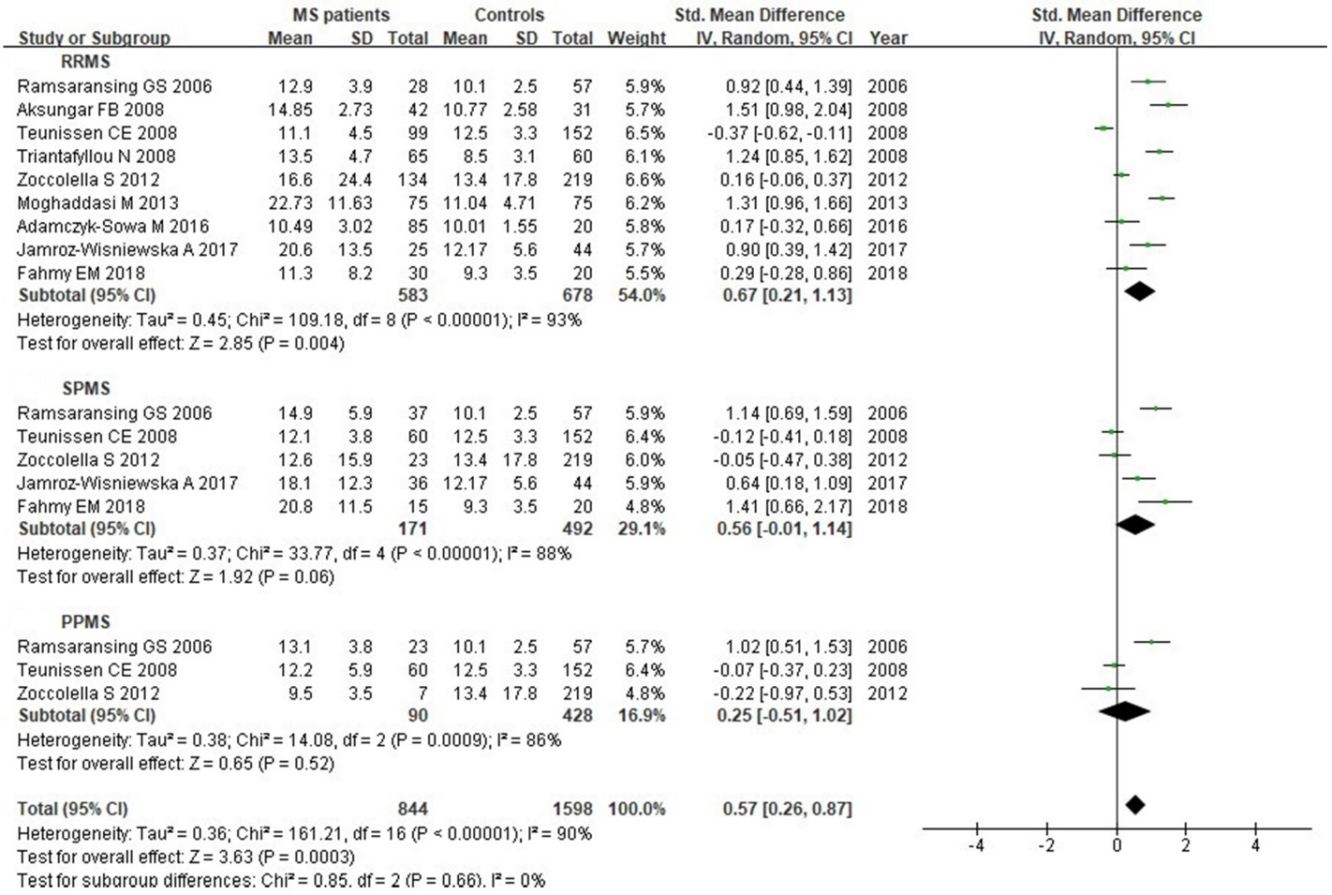
** Forest plot: subgroup analysis of Hcy serum levels between different clinical subtypes of MS patients and controls.** S*MD*: standardized mean difference; *SD*: standard deviation; Random: the random-effect model; 95% *CI:* 95% confidence interval; RRMS: relapsing-remitting multiple sclerosis; SPMS: secondary progressive multiple sclerosis; PPMS: primary progressive multiple sclerosis.

We analyzed Hcy levels according to MS clinical subtypes. There were 9 studies with a total of 1261 individuals included in this subgroup analysis. There was statistically significant difference between RRMS patients and controls, with a *SMD* of 0.67 (95% *CI*: 0.21, 1.13; *P*=0.004;* I^2^*=93%) (Fig. 8). However, there was no significant difference of Hcy serum levels between SPMS (*SMD*: 0.56, 95% *CI*: -0.01, 1.14, *P*=0.06; *I^2^*=88%) or PPMS (*SMD*: 0.25, 95% *CI*: -0.51, 1.02,* P*=0.52; *I^2^*=86%) patients and controls (Fig. 8).

**Figure 9:**
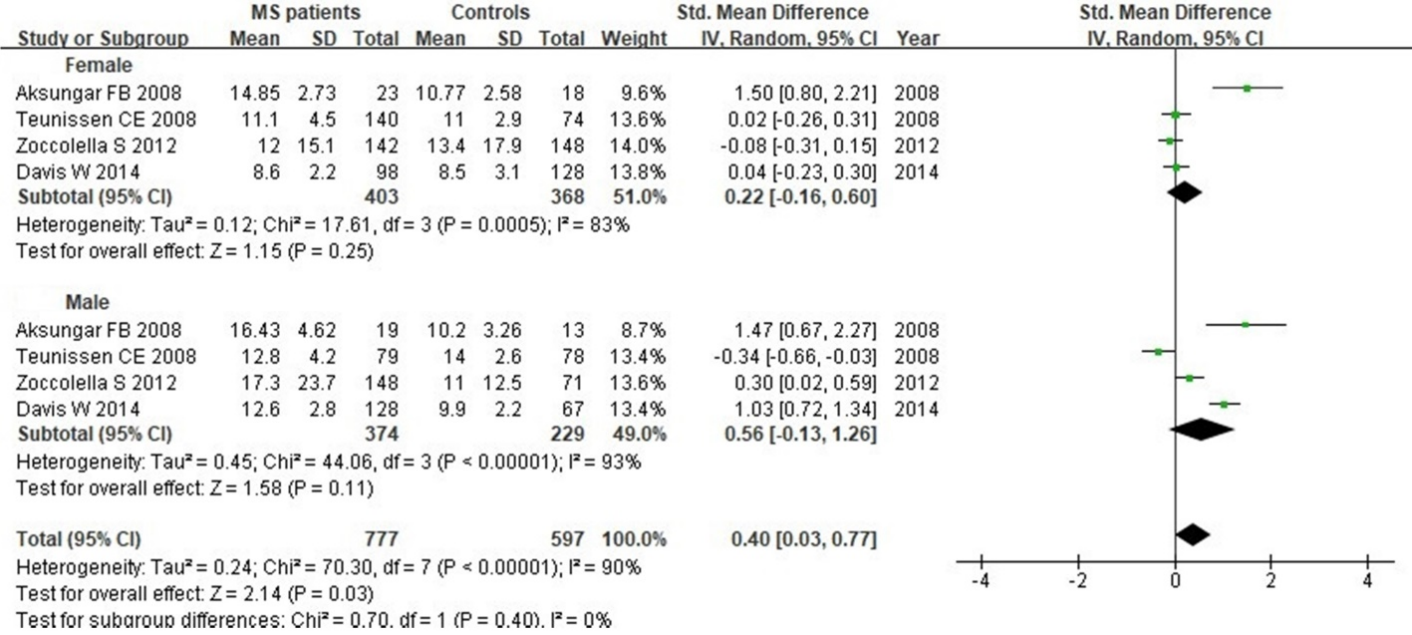
** Forest plot: subgroup analysis of Hcy serum levels between MS patients and controls according to the gender.** *SMD*: standardized mean difference; *SD*: standard deviation; Random: the random-effect model; 95% *CI*: 95% confidence interval.

We next evaluated Hcy serum levels according to the gender. A total of 1374 individuals (771 women and 603 men) from 4 studies were included in this subgroup analysis. Our results indicated that there was no significant difference when all studies were pooled in random-effect models for Hcy levels in females (*SMD*: 0.22, 95% *CI*: -0.16, 0.60, *P*=0.25) or males (*SMD*: 0.56, 95% *CI*: -0.13, 1.26,* P*=0.11) between MS patients and controls (Fig. 9).

### Sensitivity analysis

We removed 3 studies 12,30,33 which only included one subtype of MS (RRMS) to evaluate the stability of main results of associations between MS and Hcy, Vitamin B12 or folate, respectively. The results showed no significant changes obtained before and after the elimination (Table 2).

## Discussion

As a common disabling neurological disease, MS has been becoming a great challenging disorder with complex genetic and environmental interactions 4,5. It has been gradually established that there might be some close relationships between serum levels of Hcy, Vitamin B12, folate and MS 6,12,30. However, current available evidence has not been always consistent. Increased levels of Hcy combined with decreased Vitamin B12 and folate were found in Iranian RRMS patients 12. However, another study conducted in Greece inferred the opposite conclusion that circulating Hcy was not elevated in patients with MS 37. Our results combining 17 studies for Hcy were in agreement with the earlier meta-analyses 14-16, showing that MS patients were associated with elevated Hcy levels. Increased Hcy plays a role in the myelin sheath degeneration through interfering methyl group donors, causing neuroinflammation, microglial activation and other biochemical reactions in the CNS 29,42,43. Hcy can be produced during methionine metabolism, and it is metabolized either by conversion to cysteine (with Vitamin B6) or by remethylation to methionine (with Vitamin B12 and folate) 6.

Some studies suggested that lack of Vitamin B12 was related with MS due to its important role in the formation of the myelin sheath and the immunomodulatory effect 34,44. However, our study found a trend that Vitamin B12 levels and MS may be associated, to some extent, but without significant (*P*=0.23). Some prior studies showed significant difference of Vitamin B12 levels between MS patients and controls 12,14,16, while the conclusion is not further confirmed by other studies 6,15,35 and our updated meta-analysis. Our meta-analysis of Vitamin B12 showed high heterogeneity of involved studies which could have a certain impact on the negative results. In addition, some studies have explicitly restricted that subjects had not received Vitamin B12 and/or folate supplementation, while others do not control the effects of confounding factors such as diet and drugs, which may cover up the correlation between Vitamin B12 serum levels and MS. The association between folate and MS may also have the same situation.

A recent double-blinded clinical trial was performed to determine the effect of Vitamin B12 and folate supplementation on serum Hcy in RRMS patients. It has been shown that the mean levels of Hcy are significantly reduced, and both physical and mental fields of quality of life were improved in the vitamin-treated group 45. The supplementation of Vitamin B12 and folate may decrease the serum Hcy levels in MS, which would be helpful for potential treatment strategies for MS in clinical practice 45,46. However, the conclusion needs to be confirmed by conducting prospective studies with larger samples in unprecedented detail.

As for the subgroup analysis of Hcy serum levels by different MS clinical subtypes, we found statistically significant difference between RRMS patients and controls (583 RRMS and 678 controls, *P*=0.004), but no difference between SPMS patients and controls (171 RRMS and 492 controls, *P*=0.06) or PPMS patients and controls (90 RRMS and 428 controls,* P*=0.52). A recent meta-analysis evaluated the Hcy levels in two clinical phenotypes of MS patients, suggesting no significant differences between RRMS patients and controls (401 RRMS and 563 controls), or SPMS patients and controls (120 RRMS and 428 controls) 15. Different results of these two studies may due to small sample sizes, genetic factors, and environmental factors. Besides, another subgroup analysis showed increased Hcy levels in relapsing or remitting MS patients, and decreased Vitamin B12 levels in relapsing MS patients compared with controls, while no difference for folate 16. More randomized controlled trials with larger sample sizes are needed to validate the effects of Hcy on different MS clinical subtypes.

One strength of our study is evolving the most renewed literature until September 2019. We have updated 6 new studies which have been published recently in order to provide a more detailed explanation for the contribution of Hcy, Vitamin B12 and folate in the pathophysiology of MS. The updated literatures were listed as follows: five studies for Hcy 6,11,16,38,39, four studies for Vitamin B12 6,13,16,38, and four articles for folate 6,13,16,38. We thus inferred that increased Hcy levels play an important role in the pathogenesis of MS 11. Furthermore, subgroup analysis showed no statistically significant difference of Hcy levels in patients with MS between sexes.

However, there are also some limitations in our study. First, the high heterogeneity of selected studies should be taken in consideration. The heterogeneity stems from different study designs, sample sizes (varying from 45 to 436), population (gender, age, regions), and different diagnosis criteria during so many years. In this case, we utilized the random-effect model to correct the heterogeneity. Second, some other confounding factors may influence the estimating associations between Hcy, Vitamin B12, folate and MS, such as smoking, Vitamin D levels, Vitamin B complex intake, lifestyle habits, and so on. However, most prior studies did not adequately describe the relevant information. Third, the information on age, ethnicity, diet and Expanded Disability Status Scale (EDSS) scores were not taken into the subgroup analysis in our meta-analysis. Last but not the least, there were many other crucial factors which may affect the results, such as the time points of testing biomarkers and the different phases of MS. All above factors may account for the apparent discrepancies.

## Conclusion

In summary, we extend previous observations of Hcy, Vitamin B12, and folate serum levels in patients with MS. Higher circulating Hcy levels was noted in MS patients compared with controls, while no significant differences of Vitamin B12 and folate were found in this updated meta-analysis. Further subgroup analysis indicated statistically significant difference of Hcy levels between RRMS patients and controls, but not in SPMS or PPMS groups. Our study may be of main importance to elucidate the pathogenesis of MS and provide valuable information for early MS prevention. Substantial effort of functional studies is needed to explore the effects of Hcy on MS at a molecular level.

## Figures and Tables

**Table 1:** Characteristics of included studies in the meta-analysis

First author	Year	Country	MS Patients			Controls			Concentration unit
			n (M/F)	Age, y	Concentration, mean±*SD*	n (M/F)	Age, y	Concentration, mean±*SD*	
**Homocysteine**									
Vrethem M	2003	Sweden	72 (22/50)	46±13^a^	11.6±5.7^ a^	23 (7/16)	49±8^ a^	7.4±2.02^ a^	μmol/l
Besler HT	2003	Turkey	24 (16/8)	34 (31,42)^ b^	17.08±2.25^ a^	24 (16/8)	35 (32,41)^ b^	12.34±1.98^ a^	μmol/L
Ramsaransing GS	2006	Netherlands	88 (29/59)	50.09±9.32^ a,f^	13.8±4.9^ a,f^	57 (26/31)	50.86±12.8^ a^	10.1±2.5^ a^	μmol/L
Russo C	2008	Italy	94 (42/52)	36.63 ±10.36^ a^	13.19±5.58^ a^	53 (25/28)	37.15±12.06^ a^	9.81±2.53^ a^	μmol/L
Triantafyllou N	2008	Greece	65 (25/40)	39.2±8^ a^	13.5±4.7^ a^	60 (20/40)	38.2±7.7^ a^	8.5±3.1^ a^	μmol/L
Aksungar FB	2008	Turkey	42 (19/23)	42.0±4.6^ a,e^	14.85±2.73^ a,e^	31 (13/18)	30.0±5.7^ a,e^	10.77±2.58^ a,e^	μmol/L
Teunissen CE	2008	Netherlands	219 (79/140)	42.0±4.6^ a^	11.2 (8.6,14.7)^ b^	152 (78/74)	30.0±5.7^ a^	12.8 (10.2,14.6)^ b^	μmol/L
Kocer B	2009	Turkey	35 (15/20)	30.1±8.0^ a^	11.88±4.86^ a^	30 (12/18)	34.3±12.4^ a^	11.63±2.81^ a^	μmol/L
Salemi G	2010	Italy	40 (12/28)	39.5(16,58)^ b^	9.3 (4.0, 86.5)^ c^	80 (24/56)	38.5 (16,56)^ b^	6.3 (3.2,15.0)^ c^	μmol/L
Zoccolella S	2012	Italy	217 (75/142)	36.9(16,76)^ b^	9.1 (3.4,35.9)^ b^	219 (71/148)	37.5 (15,69)^ b^	8.6 (3.5,27.4)^ b^	μmol/L
Kararizou E	2013	Greece	46 (14/32)	34.2±9.2^ a^	11 (9,13)^ b^	42 (21/21)	35.8±10.3^ a^	10.8 (8.1,13.7)^ b^	μmol/L
Moghaddasi M	2013	Iran	75 (18/57)	31.97±9.05^ a^	22.73±11.63^ a^	75 (29/46)	32.33±8.86^ a^	11.04±4.71^ a^	μmol/L
Davis W	2014	South Africa	114 (16/98)	NA	9.1±2.7^ a^	195(67/128)	NA	9.0±2.9^ a^	μmol/L
Adamczyk-Sowa M	2016	Poland	102 (35/67)	42.8±10.6^ a,f^	10.29±2.92^ a,f^	20 (7/13)	34.10±11.6^ a^	10.01±1.55^ a^	μmol/L
Jamroz-Wisniewska A	2017	Poland	61 (23/38)	38.9±9.7^ a,f^	19.12±12.75^ a,f^	44 (20/24)	38±12^ a^	12.17±5.6^ a^	μmol/L
Fahmy EM	2018	Egypt	45 (23/22)	32.9±4.1^ a^	14.4±10.4^ a^	20 (NA)	NA	9.3±3.5^ a^	μmol/L
Pan L	2019	China	80 (21/59)	35.34 ± 9.25^ a^	13.39 ± 4.94^ a^	80 (28/52)	37.24 ± 8.02^ a^	8.97 ± 5.64 ^a^	μmol/L
**Vitamin B12**									
Reynolds EH	1992	England	29 (6/23)	43 (NA)	194±15^ d^	18 (NA)	33.8	253±18^ d^	pmol/L
Kira J	1994	Japan	24 (NA)	NA	629±243^ a^	21 (NA)	NA	590±262^ a^	pg/mL
Vrethem M	2003	Sweden	72 (22/50)	46±13^ a^	276±110^ a^	23 (7/16)	49±8^ a^	295±108^ a^	pmol/L
Besler HT	2003	Turkey	24 (16/8)	34±5.5^ a^	223±32^ a^	24 (16/8)	35±4.5^ a^	243±42^ a^	pmol/L
Ramsaransing GS	2006	Netherlands	88 (29/59)	50.09±9.32^ a,f^	329±374^ a,f^	57 (26/31)	50.86±12.8^ a^	466±597^ a^	pmol/L
Russo C	2008	Italy	94 (42/52)	36.63±10.36^ a^	273±112^ a^	53 (25/28)	37.15±12.06^ a^	291±109^ a^	pmol/L
Aksungar FB	2008	Turkey	42 (19/23)	42.0±4.6^ a,e^	283.45±81.56^ a,e^	31 (13/18)	30.0±5.7^ a,e^	297.56±78.63^ a,e^	pg/mL
Kocer B	2009	Turkey	35 (15/20)	30.1±8.0^ a^	221.26±72.75^ a^	30 (12/18)	34.3±12.4^ a^	257.43±105.67^ a^	pg/mL
Salemi G	2010	Italy	40 (12/28)	39.5 (16,58)^ b^	666.42±354.16^ a^	80 (24/56)	38.5 (16,56)^ b^	493.70±229.16^ a^	pg/mL
Najafi MR	2012	Iran	60 (7/53)	33±9.8^ a^	108.9±45.3^ a^	38 (5/33)	31.97±10.2^ a^	98.9±44.3^ a^	pg/mL
Zoccolella S	2012	Italy	217 (75/142)	36.9 (16,76)^ b^	508 (107,2340)^ b^	219 (71/148)	37.5 (15,69)^ b^	482 (156,2400) ^b^	pg/dL
Moghaddasi M	2013	Iran	75 (18/57)	31.97±9.05^ a^	342.64±210.66^ a^	75 (29/46)	32.33±8.86^ a^	426.48±284.65^ a^	pg/mL
Sternberg Z	2013	USA	206 (50/156)	55.6±11.5^ a^	503.4±239^ a^	142 (32/110)	56.4±13.5^ a^	666.7±314^ a^	pg/mL
Davis W	2014	South Africa	114 (16/98)	NA	628±300^ a^	195 (67/128)	NA	409±96^ a^	ng/L
Fahmy EM	2018	Egypt	45 (23/22)	32.9±4.1^ a^	170.2±223.3^ a^	20 (NA)	NA	116.2±49.3^ a^	pmol/L
Pan L	2019	China	80 (21/59)	35.34±9.25^ a^	351.18±94.44 ^a^	80 (28/52)	37.24±8.02^ a^	385.51±107.79^ a^	pg/mL
**Folate**									
Vrethem M	2003	Sweden	72 (22/50)	46±13^ a^	13.7±9.2^ a^	23 (7/16)	49±8^ a^	14.3±7.8^ a^	pmol/L
Besler HT	2003	Turkey	24 (16/8)	34 (31,42)^ b^	18.8±4.08^ a^	24 (16/8)	35 (32,41)^ b^	20.8±3.81^ a^	nmol/l
Ramsaransing GS	2006	Netherlands	88 (29/59)	50.09±9.32^ a,f^	22±35^ a,f^	57 (26/31)	50.86±12.8^ a^	24±33^ a^	nmol/L
Russo C	2008	Italy	94 (42/52)	36.63±10.3^ a^	14.3±12.7^ a^	53 (25/28)	37.15±12.06^ a^	13.8±8.2^ a^	nmol/l
Aksungar FB	2008	Turkey	42 (19/23)	42.0±4.6^ a,e^	5.56±1.75^ a,e^	31 (13/18)	30.0±5.7^ a,e^	5.68 ±1.91^ a,e^	ng/mL
Kocer B	2009	Turkey	35 (15/20)	30.1±8.0^ a^	5.11±2.10^ a^	30 (12/18)	34.3±12.4^ a^	5.97±2.15^ a^	ng/mL
Salemi G	2010	Italy	40 (12/28)	39.5 (16,58)^ b^	7.3 (2.8,25.0)^ c^	80 (24/56)	38.5 (16,56)^ b^	5.9 (1.2,16.0)^ c^	ng/mL
Zoccolella S	2012	Italy	217 (75/142)	36.9 (16,76)^ b^	5.1 (1.1,22.6)^ b^	219 (71/148)	37.5 (15,69)^ b^	5.3 (1.3,25)^ b^	mg/dL
Moghaddasi M	2013	Iran	75 (18/57)	31.97±9.0^ a^	9.74±4.77^ a^	75 (29/46)	32.33±8.86^ a^	11.64±5.76^ a^	ng/mL
Sternberg Z	2013	USA	206 (50/156)	55.6±11.5^ a^	18.2±5.8^ a^	142 (32/110)	56.4±13.5^ a^	14.8±6.0^ a^	ng/mL
Davis W	2014	South Africa	114 (16/98)	NA	19±7^ a^	195 (67/128)	NA	15±7^ a^	μg/L
Fahmy EM	2018	Egypt	45 (23/22)	32.9±4.1^ a^	4.3±6.1^ a^	20 (NA)	NA	2.2±0.5^ a^	ng/L
Pan L	2019	China	80 (21/59)	35.34±9.25^ a^	6.46±3.36 ^a^	80 (28/52)	37.24±8.02^ a^	6.74±4.77^ a^	nmol/L

MS = multiple sclerosis, M = male, F = female, *SD* = standard deviation, NA = not available. ^a^Mean±*SD*; ^b^Median (range); ^c^Median (minimum, maximum); ^d^Mean±*SE* (standard error); ^e^Combined female and male groups; ^f^Combined different MS groups.

**Table 2:** The results of sensitivity analysis about associations between MS and Hcy, Vitamin B12 or folate

Analysis	I2	Effect model	SMD	95%CI	P-value
Hcy and MS	90	Random	0.48	0.18,0.77	0.001
Vitamin B12 and MS	91	Random	-0.05	-0.35,0.25	0.75
Folate and MS	80	Random	0.12	-0.10,0.34	0.28

*SMD*: standardized mean difference; Random: the random-effect model; 95% *CI*: 95% confidence interval; Hcy: homocysteine; MS: multiple sclerosis.

## Abbreviations

CI: confidence interval
CNS: central nervous system
EDSS: Expanded Disability Status Scale
F: female
Hcy: homocysteine
M: male
MS: multiple sclerosis
NA: not available
PPMS: primary progressive multiple sclerosis
RRMS: relapsing-remitting multiple sclerosis
SD: standard deviations
SE: standard error
SMD: standardized mean difference
SPMS: secondary progressive multiple sclerosis
